# Impact characteristics of suspected concussions in elite Gaelic football and hurling: a video-based analysis

**DOI:** 10.1007/s11845-025-04163-4

**Published:** 2025-11-07

**Authors:** Ronan Davidson, Ryan McFadden, Gregory Tierney

**Affiliations:** School of Engineering, Ulster University, Belfast, UK

## Abstract

**Background:**

Sport-related concussion is a significant concern in contact sports, yet research in amateur Gaelic games is limited.

**Aims:**

To examine the characteristics and mechanisms of suspected concussions in elite Gaelic football and hurling using video-based analysis.

**Methods:**

A retrospective video analysis was conducted on 96 suspected concussions (58 Gaelic football, 38 hurling) identified from broadcast footage during the 2018–2019 inter-county seasons. Events were coded for type of play, player role, object/body part striking the head, collision direction, legality, referee penalisation, and match quarter. Descriptive statistics, Chi-square Goodness-of-Fit test and standardised residuals assessed distributions.

**Results:**

In Gaelic football, suspected concussions were evenly distributed across quarters, χ²=6.23, *p* = 0.101. In hurling, incidents were overrepresented in the fourth quarter, χ²=14.00, *p* = 0.003, residual = + 3.08. Tackles were significantly more frequent in football (residual = + 8.63, χ²=95.79, *p* < 0.001) and hurling (residual = + 4.14, χ²=28.58, *p* < 0.001), while off-the-ball collisions and goalpost impacts were less frequent. Ball carriers were overrepresented in football (residual = + 5.99, χ²=48.38, *p* < 0.001) and hurling (residual = + 3.41, χ²=16.32, *p* = 0.001). Arms were the main impact source in football (residual = + 8.08, χ²=79.93, *p* < 0.001) with shoulders/torsos in hurling (residual = + 4.24/+2.87, χ²=36.53, *p* < 0.001). Front-on collisions predominated in both codes. Illegal play was significant in football (residual = + 2.6, χ²=13.52, *p* < 0.001), with illegal incidents penalised (residual = + 3.34, χ²=22.35, *p* < 0.001).

**Conclusion:**

The findings underscore the need for targeted rule enforcement, player education, and management to reduce concussion risk in Gaelic football and hurling.

## Introduction

Sport-related concussion (SRC), a form of mild traumatic brain injury (mTBI), has increasingly been recognised as a major public health concern across contact and collision sports [1]. Defined by the International Consensus Conference on Concussion in Sport as a change in brain function caused by biomechanical forces, SRC may be associated with both short-term neurological symptoms and potential long-term consequences, including chronic traumatic encephalopathy (CTE), Alzheimer’s disease, and other neurodegenerative conditions [2]. While substantial progress has been made in professional sports such as American football, rugby, and ice hockey to address the risks of concussion, comparatively little attention has been paid to amateur and regionally concentrated sports, including those governed by the Gaelic Athletic Association (GAA) [3].

The GAA is Ireland’s largest sporting organisation and is unique in its role as both a cultural institution and an amateur sporting body [3]. Two of its most popular codes, Gaelic football and hurling, are fast and physically demanding field sports. Gaelic football is played by teams of 15 athletes on a grass pitch [4–6]. Players advance a round ball through kicking, hand passing, or carrying, with physical contact allowed primarily through shoulder-to-shoulder challenges. Mouthguards are compulsory, but helmets are not worn. Hurling, in contrast, is played with a small hard ball (sliotar) and a wooden stick (hurley) and is frequently referred to as the fastest field game globally [4–6]. Players wear helmets with faceguards. Shoulder-to-shoulder collisions are also permitted, and the pace of play can give rise to unanticipated head contact [4–6].

Epidemiological surveys among GAA athletes highlight the burden of concussion across both codes [7, 8]. One study reported that more than half of players had experienced at least one concussion, with a significant proportion reporting multiple concussions [8]. Yet many of these cases remain undiagnosed, reflecting limitations in awareness, sideline management, and access to medical resources at the point of injury [4–6]. In response, the GAA has introduced Concussion Management Guidelines based on international consensus and more recently implemented a concussion interchange rule to facilitate in-game medical assessment [4–6]. Despite these advances, challenges persist in ensuring that suspected concussions are identified, assessed, and managed appropriately. Evidence from other sports underscores the risks associated with inadequate concussion recognition, including long-term cognitive decline linked to repeated head trauma [9].

Previous video-analysis studies on elite hurling and Gaelic football reported that potential concussive events (PCEs) are frequent and often involve visible head impacts [3–6]. However, limited research has examined the characteristics of concussion incidents across both Gaelic football and hurling. Given their fast pace and frequent collisions, these sports provide unique contexts in which to study concussion mechanisms. Understanding how suspected concussions occur in Gaelic games may offer critical insights for player protection strategies and targeted rule modifications. The present study addresses this gap by conducting a video-based analysis of suspected concussions in Gaelic football and hurling during the 2018 and 2019 inter-county seasons. By comparing the characteristics of incidents across both sports, the study aims to identify mechanisms most associated with visible concussion signs and provide evidence to inform concussion prevention and management within the GAA.

## Methods

### Study design and data source

This study employed a retrospective video-based analysis of suspected concussions in elite-level Gaelic football and hurling. The dataset comprised cases previously identified in two published video-analysis studies of Gaelic games, which together reported potential concussive events (PCEs) across inter-county competitions during the 2018 and 2019 seasons [3–6]. From these datasets, we extracted only those incidents in which players demonstrated consensus-agreed visible signs and symptoms of concussion [10, 11]. These signs included lying motionless, motor incoordination, impact seizure, tonic posturing, no protective action, floppy and blank/vacant look [10, 11].

In total, 96 suspected concussions were included in the present study: 58 from Gaelic football and 38 from hurling. All incidents were captured from broadcast match footage provided by national television coverage. The study received ethical approval from the Ulster University Faculty of Computing, Engineering and the Built Environment Ethics Committee (Ref #: CEBE-25–2728).

### Video analysis and coding protocol

Each suspected concussion was reviewed in detail using QuickTime Player (v10.5), which enabled frame-by-frame analysis of the moments leading up to, during, and immediately following the impact. A trained reviewer independently coded all incidents [12]. Incidents were coded according to the variables in Table 1.

**Table 1:** Video analysis coding framework

Variable	Categories
Type of play	Tackle, Shoulder challenge, Aerial Contest, Off-the-ball, Goalpost Impact
Player role	Ball carrier, Tackler, 50/50 contest, Loose ball, Other
Object/body part striking head	Shoulder, Arm, Torso/hip, Ground, Leg, Hurley, Sliotar/ball, Teammate, Head
Collision Direction	Front-on, Side-on, From Behind
Legality of Contact	Legal, Illegal
Referee penalisation	Penalised, Not Penalised
Match Quarter	Q1, Q2, Q3, Q4

### Statistical analysis

Descriptive statistics were used to summarise the frequency of suspected concussions across each variable for Gaelic football and hurling separately. Chi-square Goodness-of-Fit tests were employed to examine whether the observed distributions within each variable deviated significantly from a uniform distribution [13]. Statistical significance was set at *p* < 0.050. Standardised residuals were examined to identify which specific categories contributed most to the deviation from the expected distribution. Standardised residuals greater than + 1.96 or less than − 1.96 were interpreted as indicating that a category occurred significantly more or less frequently than expected, respectively. The analyses were performed using Python (Version 3.11) with the SciPy statistical library.

## Results

### Match quarters

**Fig. 1:**
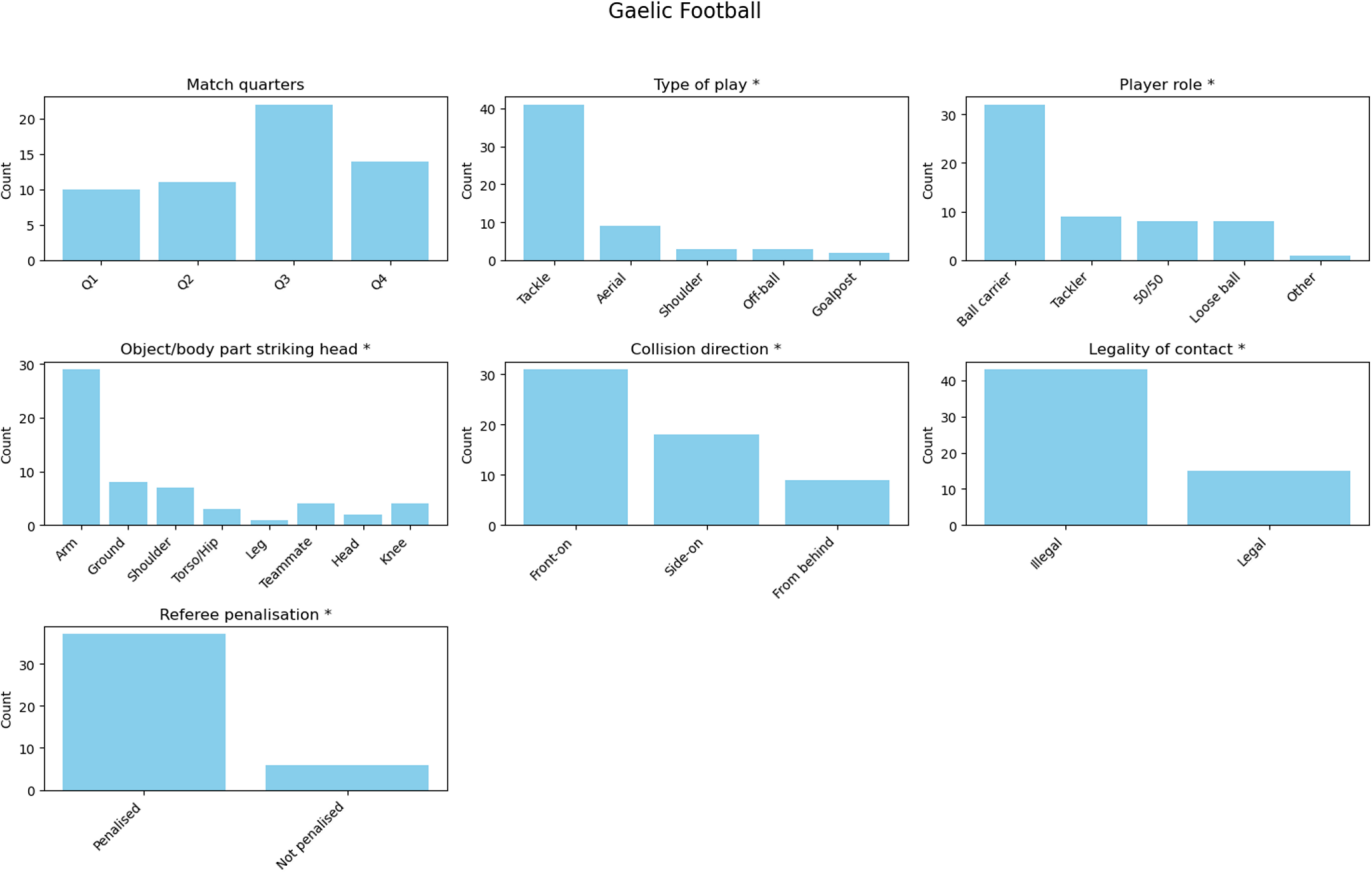
Distribution of suspected concussion events in Gaelic football across different match characteristics. An asterisk (*) in the subplot title indicates *p* < 0.050

In Gaelic football (Fig. 1), the distribution of suspected concussions across match quarters did not differ significantly from a uniform distribution, χ²= 6.23, *p* = 0.101. Standardised residuals indicated that incidents were most frequent in the third quarter (residual = + 2.05), though this did not reach statistical significance.

**Fig. 2:**
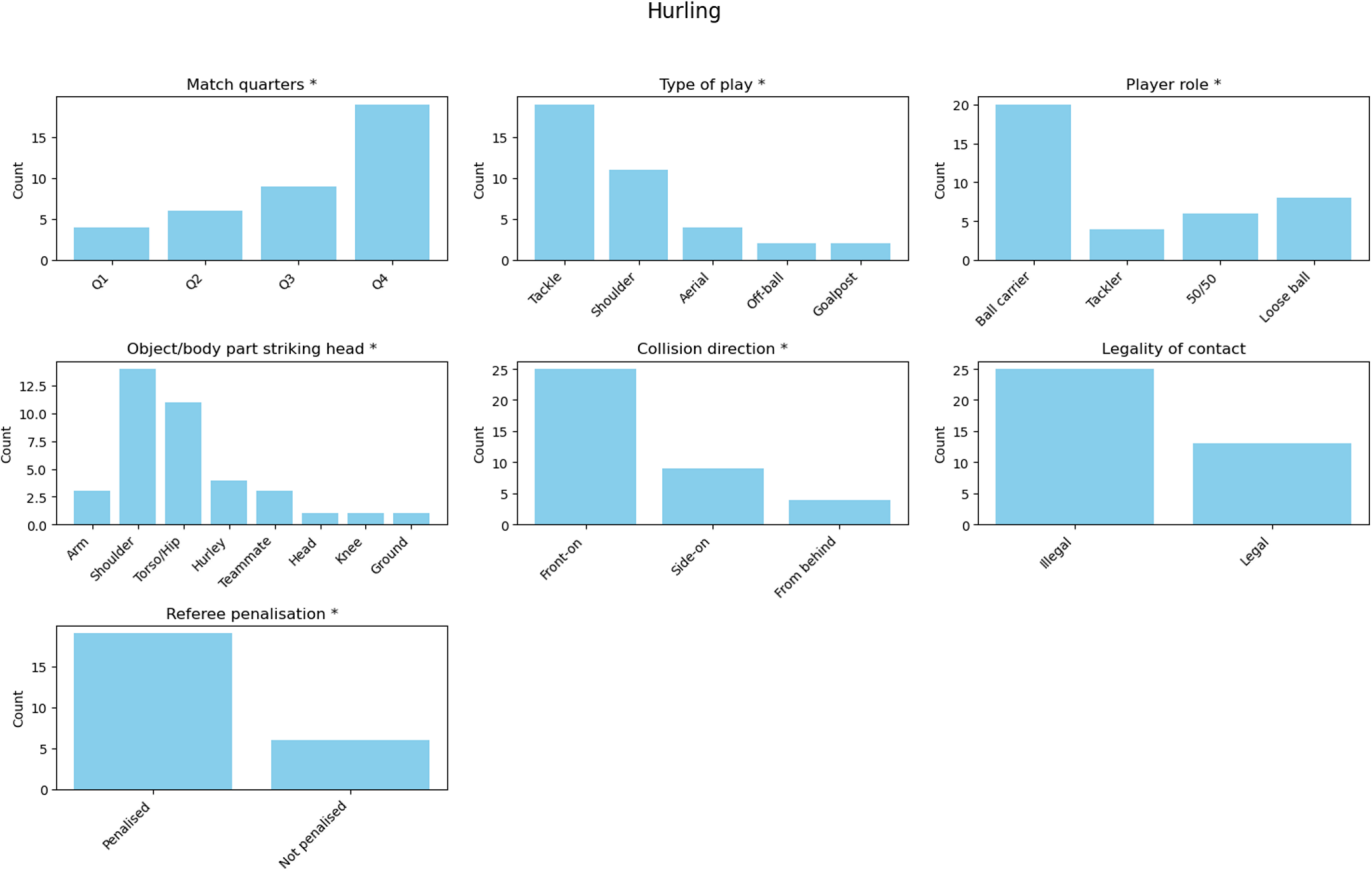
Distribution of suspected concussion events in Hurling across different match characteristics. An asterisk (*) in the subplot title indicates *p* < 0.050

In contrast, hurling (Fig. 2) displayed a significant deviation from uniformity, χ² = 14.00, *p* = 0.003, with substantially more suspected concussions occurring in the fourth quarter than expected (residual = + 3.08, χ² = 14.00, *p* = 0.003).

### Type of play

In Gaelic football, tackles accounted for significantly more suspected concussions than expected (residual = + 8.63, χ² = 95.79, *p* < 0.001), while shoulder challenges (residual = − 2.53, χ² = 95.79, *p* < 0.001), off-the-ball collisions (residual = − 2.53, χ² = 95.79, *p* < 0.001), and goalpost impacts (residual = − 2.82, χ² = 95.79, *p* < 0.001) were significantly less frequent.

Similarly, in hurling, tackles occurred significantly more often than expected (residual = + 4.14, χ² = 28.58, *p* < 0.001), whereas off-the-ball collisions (residual = − 2.03, χ² = 28.58, *p* < 0.001) and goalpost impacts (residual = − 2.03, χ² = 28.58, *p* < 0.001) occurred less frequently.

### Player role

In Gaelic football, ball carriers were involved in significantly more incidents than expected (residual = + 5.99, χ² = 48.38, *p* < 0.001), while incidents classified as “other” were significantly less frequent (residual = − 3.11, χ² = 48.38, *p* < 0.001).

In hurling, ball carriers were again significantly over-represented (residual = + 3.41, χ² = 16.32, *p* = 0.001).

### Object or body part striking the head

In Gaelic football, opponents’ arms were responsible for significantly more head contacts than expected (residual = + 8.08, χ² = 79.93, *p* < 0.001), whereas impacts from legs occurred less frequently (residual = − 2.32, χ² = 79.93, *p* < 0.001).

In hurling, impacts from opponents’ shoulders (residual = + 4.24, χ² = 36.53, *p* < 0.001) and torsos/hips (residual = + 2.87, χ² = 36.53, *p* < 0.001) were significantly higher than expected.

### Collision direction

In Gaelic football, front-on collisions occurred significantly more often than expected (residual = + 2.65, χ² = 12.66, *p* = 0.002), while collisions from behind occurred significantly less often (residual = − 2.35, χ² = 12.66, *p* = 0.002).

A similar pattern was observed in hurling, where front-on impacts were significantly over-represented (residual = + 3.47, χ² = 19.00, *p* < 0.001) and those from behind were significantly under-represented (residual = − 2.44, χ² = 19.00, *p* < 0.001).

### Legality of contact and referee penalisation

In Gaelic football, suspected concussions were significantly more likely to arise from illegal play, χ²= 13.52, *p* < 0.001, In hurling, the difference was not statistically significant, χ² = 3.79, *p* = 0.052.

The likelihood of referee penalisation following an illegal incident differed significantly from a uniform distribution in Gaelic football (χ² = 22.35, *p* < 0.001) and hurling (χ² = 6.76, *p* = 0.009).

## Discussion

### General

This study examined the impact characteristics of suspected concussions in elite Gaelic football and hurling using video-based analysis. By focusing on incidents in which players displayed visible concussion signs, the study provides insight into how suspected concussions occur in these two culturally significant but under-researched sports. The findings reinforce the central role of tackles, frontal impacts, and ball carriers as high-risk contexts, while also revealing code-specific patterns such as the predominance of arm-led impacts in Gaelic football and shoulder or torso collisions in hurling. These results both confirm and extend previous work on Gaelic games and place them in broader context with global concussion research.

### Timing of suspected concussions

In Gaelic football, suspected concussions appeared most frequently in the third quarter, although this distribution did not reach statistical significance. In hurling, by contrast, incidents were significantly overrepresented in the final quarter, with half of all suspected concussions occurring in this period. The significance of this late-game concentration in hurling is notable, as it echoes findings from professional rugby union, where higher suspected concussion rates have been reported during later stages of matches [12].

One plausible explanation is fatigue-related impairment [14]. Fatigue has been linked to declines in neuromuscular control, slower reaction times, and impaired decision-making, all of which increase vulnerability to head impacts [14]. Rotundo et al. [6] analysing potential concussive events in hurling, also reported increased fourth-quarter incidence, suggesting this may be a consistent feature of the sport. The high-intensity, continuous nature of hurling with rapid transitions, repeated sprints, and aerial contests may accelerate fatigue accumulation. Gaelic football did not show a significant late-game effect which may reflect greater variability in match tempo and tactical patterns. Regardless, these results suggest that conditioning, substitutions [15], and awareness of fatigue-related risks may be particularly important in hurling.

### Type of play and player role

Tackling emerged as the dominant context for suspected concussions in both sports, with highly significant distributions. In Gaelic football, the majority of incidents occurred during tackles, a concentration similar to rugby union, where 70–80% of concussions are tackle-related [16–18]. Tackles involve close body proximity, contact with arms or shoulders and rapid speeds, creating multiple avenues for concussion.

The role of the injured player further reinforces the centrality of tackling. Ball carriers were significantly overrepresented in both sports, accounting for most incidents in Gaelic football and hurling. This contrasts rugby, where tacklers are frequent concussion victims [16–18]. Carrying the ball imposes both cognitive and physical demands, limiting a player’s ability to anticipate or brace for impact. The heightened risk for ball carriers in Gaelic games highlights the need for coaching emphasis on safe ball-carrying strategies, communication from teammates, and tactical support to reduce player-to-player contact.

### Mechanisms of impact

The analysis of impact sources revealed distinct contrasts between the two sports. In Gaelic football, the arm was the predominant contact point, followed by the ground and shoulders. This suggests that swinging or flailing arms during tackles are a major source of head contact. Such mechanisms are less prominent in rugby, where structured tackling techniques are more heavily coached and regulated [19].

In hurling, shoulders and torsos or hips dominated. Rotundo et al. [6], similarly found that hurley stick impacts were relatively infrequent compared with body collisions. The prominence of shoulder-led contacts highlights the cultural importance of the shoulder challenge in hurling, but also its potential danger. Importantly, both studies challenge the perception that stick-related impacts are the principal danger in hurling. While hurleys did cause some suspected concussions, body contact was the primary mechanism. Previous rugby analyses suggest that shoulder-to-head contact is among the most concussive impact types due to tackle styles and the large effective mass involved [16–18]. By analogy, shoulder collisions in hurling may represent a critical intervention point for rule enforcement and coaching technique.

### Collision direction

Both sports showed a statistically significant predominance of front-on collisions. This is similar to findings in American football and rugby [20, 21]. In Gaelic games, the attacking structure frequently places advancing ball carriers in direct confrontation with defenders, predisposing players to frontal impacts.

Front-on collisions are particularly concerning because they often occur when players are upright or looking down at the ball, reducing their ability to prepare for impact. Moreover, they can produce substantial linear and rotational accelerations due to high closing velocities [22]. The predominance of frontal mechanisms highlights the need for both tactical awareness and referee vigilance in monitoring late or high front-on hits.

### Legality of contact

A majority of suspected concussions arose from illegal play in both sports, though the strength of this finding differed. In Gaelic football, 74% of incidents were classified as illegal and the distribution was statistically significant (*p* < 0.001). In hurling, 66% of incidents were illegal, but this result narrowly missed significance (*p* = 0.052). Thus, while there appears to be a trend towards illegal play in both sports, conclusive evidence was only observed in football.

The prevalence of illegal play aligns with Rotundo et al. [6], who identified frequent fouls associated with head contact in hurling, and with Sokol-Randell et al. [5], who observed similar patterns in football. Both studies highlight that illegal collisions whether late tackles, high shoulders, or reckless stick use remain a persistent cause of head trauma in Gaelic games.

### Referee penalisation

Encouragingly, most illegal contacts resulting in suspected concussions were penalised. These proportions were statistically significant in both sports, demonstrating that referees are generally effective at identifying and sanctioning dangerous play. However, nearly one in four illegal hurling incidents went unpenalised. This aligns with concerns raised by Rotundo et al. [6], who noted that officiating consistency in hurling may lag behind football.

Increased referee education, the involvement of additional sideline officials, and possible use of video replay at elite levels could help close this gap [23, 24]. The GAA’s introduction of a concussion interchange rule demonstrates willingness to innovate in this area, and officiating standards represent another potential avenue for concussion mitigation [3–6].

### Comparison between Gaelic football and hurling

The present study highlights both commonalities and sport-specific nuances in concussion mechanisms. Tackles and frontal impacts emerged as consistent contexts across both sports. However, the specific sources of impact diverged. Arms were dominant in football, while shoulders and torsos led in hurling. Similarly, only hurling showed a significant late-game concentration of incidents.

These contrasts illustrate how rules, equipment, and style of play shape concussion risk. Gaelic football’s emphasis on hand passing may contribute to the prevalence of arm-led head impacts. Hurling’s shoulder challenge tradition and stick use create different collision dynamics. Both sports demonstrated that helmets (in hurling) and mouthguards (in football) alone are insufficient in preventing suspected concussions [25–27].

### Implications for the GAA

The findings carry several implications for player safety. First, tackling technique requires greater emphasis in coaching curricula. Structured instruction on safe head placement, controlled engagement, and avoidance of swinging arms could reduce concussion risk. Second, referee training should continue to prioritise the identification and sanctioning of high-risk contacts, particularly late or frontal hits. Third, player education around fatigue management and risk awareness may help mitigate fourth-quarter vulnerability in hurling. Rule enforcement and cultural attitudes towards physical contact must evolve [7]. In football, stricter regulation of arm-led tackles could be considered, while in hurling, reevaluating the conditions under which shoulder challenges are deemed legal may be warranted.

## Limitations

Several limitations should be noted. Broadcast footage restricts the angles and clarity of analysis, and concussion identification relied solely on visible signs rather than medical confirmation similar to previous studies [12, 18]. The dataset was limited to two seasons, constraining sample size, particularly for hurling. Though the main characteristics of Gaelic football and Hurling remain the same, the GAA has implemented rule changes since the 2018/19 season, and thus the findings should be interpreted within this context. Additionally, only male elite-level inter-county players were included, leaving women’s Gaelic games and youth participation unexplored. Only a single rater was utilised in the current study. Multiple raters and high inter-rater agreement may have strengthened the findings. Future research should expand the dataset across multiple years, incorporate women’s competitions, and combine video analysis with clinical follow-up. The integration of instrumented mouthguards, which are increasingly used in rugby [28], could provide biomechanical confirmation of head kinematics during suspected concussions.

## Conclusion

This video-analysis study demonstrates that suspected concussions in Gaelic football and hurling arise predominantly from tackles and frontal collisions, with ball carriers consistently susceptible. While both sports share common mechanisms, sport-specific tendencies were also observed. Arm-led impacts in football, shoulder and torso collisions in hurling, and a late-game concentration of incidents in hurling only. Illegal play was strongly implicated in football, while referees penalised most dangerous contacts in both codes. These findings reinforce the need for targeted interventions in the GAA, including enhanced coaching on tackle technique, stricter officiating of high-risk contacts, and player education on fatigue-related vulnerability. By addressing these areas, the GAA can strengthen concussion prevention and management in its most popular codes, protecting athletes while preserving the cultural and sporting significance of Gaelic games.

## References

[1] Tierney G (2024) Concussion biomechanics, head acceleration exposure and brain injury criteria in sport: a review. Sports Biomech 23:1888–1916 34939531 10.1080/14763141.2021.2016929

[2] Patricios JS, Schneider KJ, Dvorak J et al (2023) Consensus statement on concussion in sport: the 6th International Conference on Concussion in Sport–Amsterdam, October 2022. Br J Sports Med 57(11):695–711 37316210 10.1136/bjsports-2023-106898

[3] Sokol-Randell D, Rotundo MP, Tierney G et al (2021) Frequent but limited assessment of potentially concussed players in Gaelic football: an opportunity to learn from other sports. Ir J Med Sci 190:787–792 32997230 10.1007/s11845-020-02390-5

[4] Sokol-Randell D, Rotundo MP, Tierney G et al (2022) Video analysis of potential concussions in elite male hurling: are players being assessed according to league guidelines? Ir J Med Sci 191:2335–2342 34664223 10.1007/s11845-021-02798-7 PMC8523202

[5] Sokol-Randell D, Rotundo MP, Tierney G et al (2021) Characteristics of potential concussive events in elite male Gaelic football players: a descriptive video-analysis. J Sports Sci 39:1700–1708 33722171 10.1080/02640414.2021.1896455

[6] Rotundo MP, Sokol-Randell D, Bleakley C et al (2023) Characteristics of potential concussive events in elite hurling: a video-analysis study. Ir J Med Sci 192:3175–3185 36800054 10.1007/s11845-023-03307-8 PMC10692028

[7] Daly E, Ryan L (2024) Concussion management and concussion recovery in Gaelic games: a qualitative analysis. Front Sports Act Living 6:1470358 39398267 10.3389/fspor.2024.1470358 PMC11466752

[8] O’Connor S, Moran K, Burke C et al (2019) Sports-related concussion in adolescent Gaelic games players. Sports Health 11:498–506 31592720 10.1177/1941738119875978 PMC6822212

[9] Cunningham J, Broglio S, Wilson F (2018) Influence of playing rugby on long-term brain health following retirement: a systematic review and narrative synthesis. BMJ Open Sport Exerc Med 4:e000356 29719729 10.1136/bmjsem-2018-000356 PMC5926651

[10] Davis GA, Makdissi M, Bloomfield P et al (2019) International study of video review of concussion in professional sports. Br J Sports Med 53:1299–1304 30262454 10.1136/bjsports-2018-099727

[11] Davis GA, Makdissi M, Bloomfield P et al (2019) International consensus definitions of video signs of concussion in professional sports. Br J Sports Med 53:1264–1267 30954947 10.1136/bjsports-2019-100628

[12] Tierney GJ, Lawler J, Denvir K et al (2016) Risks associated with significant head impact events in elite rugby union. Brain Inj 30:1350–1361 27715327 10.1080/02699052.2016.1193630

[13] Brustio PR, Kelly AL, Lupo C et al (2022) The influence of contextual factors on the relative age effect in male international rugby union: the impact of sociocultural influences and playing position. Children (Basel) 9:1941 36553385 10.3390/children9121941 PMC9777006

[14] Gabbett TJ (2008) Influence of fatigue on tackling technique in rugby league players. J Strength Cond Res 22:625–632 18550983 10.1519/JSC.0b013e3181635a6a

[15] Allan D, Tooby J, Starling L et al (2024) Player and match characteristics associated with head acceleration events in elite-level men’s and women’s rugby union matches. BMJ Open Sport Exerc Med 10:e001954 39381414 10.1136/bmjsem-2024-001954 PMC11459297

[16] Tucker R, Raftery M, Fuller GW et al (2017) A video analysis of head injuries satisfying the criteria for a head injury assessment in professional rugby union: a prospective cohort study. Br J Sports Med 51:1147–1151 28663217 10.1136/bjsports-2017-097883

[17] Tucker R, Raftery M, Kemp S et al (2017) Risk factors for head injury events in professional rugby union: a video analysis of 464 head injury events to inform proposed injury prevention strategies. Br J Sports Med 51:1152–1157 28642222 10.1136/bjsports-2017-097895

[18] Tierney GJ, Denvir K, Farrell G et al (2017) The effect of tackler technique on head injury assessment risk in elite rugby union. Med Sci Sports Exerc 50:603–608 10.1249/MSS.0000000000001461 29049096

[19] Hendricks S, Lambert M (2010) Tackling in rugby: coaching strategies for effective technique and injury prevention. Int J Sports Sci Coach 5:117–136

[20] Burger N, Lambert MI, Viljoen W et al (2016) Tackle technique and tackle-related injuries in high-level South African rugby union under-18 players: real-match video analysis. Br J Sports Med 50:932–938 26781294 10.1136/bjsports-2015-095295

[21] Sherwood CP, Grogan F, McMurry TL et al (2025) Tackle techniques and characteristics associated with a concussion in tackling players in the National Football League. Am J Sports Med 53:1142–1151 40037391 10.1177/03635465251321005

[22] Tierney GJ, Tucker R (2022) The role of player mass and contact speed on head kinematics and neck dynamics in rugby union tackling. Scand J Med Sci Sports 32(2):298–312 34741337 10.1111/sms.14090

[23] Mitchell SL, Tierney GJ (2023) An assessment of the world rugby law application guidelines for the breakdown on sanctioning and player adherence. Int J Sports Sci Coach 18(3):883–888

[24] Mitchell S, Tierney GJ (2021) Sanctioning of breakdown infringements during the knockout stage of the 2019 Rugby World Cup. Int J Sports Sci Coach 16:407–414

[25] McIntosh AS, McCrory P, Finch CF et al (2009) Does padded headgear prevent head injury in rugby union football? Med Sci Sports Exerc 41:306–313 19127196 10.1249/MSS.0b013e3181864bee

[26] Kemp SP, Hudson Z, Brooks JH et al (2008) The epidemiology of head injuries in English professional rugby union. Clin J Sport Med 18:227–234 18469563 10.1097/JSM.0b013e31816a1c9a

[27] Naunheim RS, Ryden A, Standeven J et al (2003) Does soccer headgear attenuate the impact when heading a soccer ball? Acad Emerg Med 10:85–90 12511322 10.1111/j.1553-2712.2003.tb01983.x

[28] Allan D, Tooby J, Starling L et al (2024) Head kinematics associated with off-field head injury assessment (HIA1) events in a season of English elite-level club men’s and women’s rugby union matches. Sports Med. 10.1007/s40279-024-02146-6 39549223 10.1007/s40279-024-02146-6 PMC12106129

